# The Media and Sexual Violence Among Adolescents: Findings from a Qualitative Study of Educators Across Vietnam

**DOI:** 10.1007/s10508-024-02869-7

**Published:** 2024-05-10

**Authors:** Katherine M. Anderson, Alicia Macler, Irina Bergenfeld, Quach Thu Trang, Kathryn M. Yount

**Affiliations:** 1 Department of Behavioral, Social, and Health Education Sciences, Rollins School of Public Health, Emory University, Atlanta, GA USA; 2 Hubert Department of Global Health, Rollins School of Public Health, Emory University, 1518 Clifton Rd NE, Atlanta, GA 30322 USA; 3 Center for Creative Initiatives in Health and Population, Hanoi, Vietnam; 4 Department of Sociology, Emory University, Atlanta, GA USA

**Keywords:** Sexual violence, Youth, Media, Violence prevention, Vietnam

## Abstract

Growing access to technology and media has presented new avenues of influence on youth attitudes and norms regarding sexuality and sexual violence, as well as new technological pathways through which to perpetrate sexual violence. The aim of this research was to understand contextual influences on and needs for scale-up of sexual violence prevention programming in the media-violence context of Vietnam. We conducted 45 interviews with high school teachers (*n* = 15), university lecturers (*n* = 15), and affiliates from youth-focused community service organizations (*n* = 15) from across Vietnam. Additionally, we conducted four sector-specific focus groups with a sub-sample of interview participants (*k* = 4, *n* = 22). Media and technology were brought up consistently in relation to sexual violence prevention and sexual health information. Key informants noted that, in Vietnam, generational differences in acceptability of sex and lack of comprehensive sexuality education intersect with new technological opportunities for exposure to sexual information and media. This creates a complex landscape that can promote sexual violence through priming processes, instigate mimicry of violent media, and presents new opportunities for the perpetration of sexual violence though technology. Development of comprehensive sexual education, including violence prevention education, is imperative, with consideration of age-specific needs for Vietnamese youth.

## Introduction

### Prevalence of Sexual Violence Globally in Asia/Pacific and in Vietnam

Sexual violence, defined as a sexual act committed or attempted in the absence of freely given consent, is a worldwide public health challenge (Basile et al., 2014). Sexual violence includes contact-based acts, such as non-consensual sexual intercourse, and non-contact-based acts, such as sexual harassment, unwanted exposure to sexual situations, and non-consensual filming and/or dissemination of explicit photographs (Basile et al., 2014). Globally, at least 35.6 percent of all women ages 15 years or older have reported experiencing sexual violence (World Health Organization (WHO), 2021). Men also may experience sexual violence; however, the global prevalence of exposure to sexual violence for men is much lower than for women (Institute for Health Metrics and Evaluation [IHME], 2017). Moreover, men are shown to perpetrate the majority of instances of sexual violence (James-Hawkins et al., 2019). Survivors of sexual violence are at heightened risk of experiencing immediate and long-term negative physical and mental health outcomes, as well as adverse social outcomes, such as diminished academic achievement (Amar & Gennaro, 2005; Fielding-Miller et al., 2021; Gonzales et al., 2005).

Southeast Asia has a high lifetime prevalence of physical or sexual violence by a partner or sexual violence by a non-partner; over 34% of women ages 15 to 49 in Southeast Asia report at least one of these experiences (WHO, 2021). In select countries of the Asia–Pacific region, 24.3% of men reported ever perpetrating sexual partner violence, and 10.9% of men had perpetrated non-partner rape in their lifetime (Fulu et al., 2013). Of men who reported non-partner rape, 57.6% reported perpetrating rape more than one time (Fulu et al., 2013). In Vietnam, sexual violence remains understudied and likely under-reported, though the available data suggest that sexual violence victimization is widespread (Pham, 2015; Winzer et al., 2019). According to the 2019 Vietnam survey of violence against women, 13.3% of women reported ever experiencing sexual violence by a husband or partner, and 9.0% reported ever experiencing sexual violence by a non-partner (MOLISA & UNFPA, 2020). However, other estimates gauge sexual violence victimization as more pervasive, with almost 20% of women 20 to 24 years reporting sexual violence victimization since the age of 15 (Le et al., 2019). Across age groups, adolescent women 15–19 years and young women 20–24 years are reported to experience the highest and second highest rates of non-partner sexual violence since age 15, at 24% and 17%, respectively (MOLISA & UNFPA, 2020).

### Theories of Media and Violence

Several theories exist to explain how media influences cognition and behavior (Valkenburg et al., 2016), with variation by context, modality of the media, and predisposing personal factors. These include routine activity theory, which posits that individuals can be motivated to enact violence or another crime when an available victim, an offender, and the absence of a protective force or guardian for the potential victim converge (Aizenkot, 2022; Kumar et al., 2021; Madero-Hernandez & Fisher, 2012; Räsänen et al., 2016; Van Ouytsel et al., 2018) and social ecology theory, which emphasizes the role of the social environment (Lou et al., 2012; Stokols, 1992).

For purposes of the current analysis, we are informed by a combination of theories that conceptually overlap and build upon each other, including mimicry, sexual script theory, social cognitive theory, and cultivation theory. In the immediate aftermath of media exposure to violence, enacted violence is thought to operate through priming processes, arousal processes, and mimicking behaviors (Huesmann, 2007). The third of these, mimicry, is the imitation of behaviors seen in media (Huesmann, 2007), and is a common theme in theories of violence. Sexual script theory embraces the concept of mimicry by asserting that individuals model their sexual expectations, norms, desires, and decisions after portrayals of sex in their culture, such as in media (Wiederman, 2015), while social cognitive theory and social learning theory employ the similar concept of observational learning (Bandura, 2001; Bandura & Walters, 1977). Both social cognitive theory and social learning theory have been applied to the context of media, sexual activity, and violence (Brem et al., 2021; Hedrick, 2021; Hust et al., 2019; Marshall et al., 2021; Sun et al., 2016; Walker, 2021). Youth employ observational learning regularly as part of development (Fryling et al., 2011), including in cases of observed violence (Flannery et al., 2007). Observational learning typically occurs over time (Bandura, 2008), with replication of observed behaviors lasting well beyond the observed event (Fryling et al., 2011). According to cultivation theory, which also has been applied to media-violence research (Hedrick, 2021; Moorman, 2022), greater and repeated use of media is associated with greater acceptance of the norms and beliefs conveyed by that media (Morgan et al., 2014). Compounding this, repeated observation of arousing content can, in turn, cause desensitization, in which negative reactions to events like violence can become dampened over time, allowing viewing or participation in violence without negative affect (Huesmann, 2007). Notably, these theories lack significant consideration of youth as critical thinkers, with the assumption that they absorb witnessed behaviors with limited processing prior to reenactment. Literature supports more complex processes, such as emotion regulation and rumination (Brimmel et al., 2023; Felix et al., 2022; McComb & Mills, 2021), which we acknowledge and consider in our theoretical approach.

### Pathways to Sexual Knowledge and Violence: Media Exposure and Use

Paradoxically, the global rise of technological connectivity has created new pathways to access sexual information and to execute sexual violence, the impacts of which may not be fully captured by existing data. Technology increasingly is used to access mass media sources, including informational media, such as news and informational websites, social media, such as social networking sites, or entertainment media, such as films. Most adolescents and young adults use the internet for health information-seeking (Buhi et al., 2009; Santor et al., 2007), with sexual health searched more often than any other health topic (Buhi et al., 2009). For many, the internet is the primary source of information, even with recognition that schools and medical professions may be better sources of information (Shih et al., 2015).

In Southeast Asia, an estimated 17% of individuals have used media to learn about sex (Gesselman et al., 2020). This gathering of information may lead to positive, neutral, or negative information about sex, to the extent that informational media and entertainment media may contribute indirectly to sexually violent behaviors, wherein content may reinforce harmful normative beliefs about sexual violence and may normalize or even promote contact- and non-contact forms of sexually violent behavior. This is aligned with theory of priming processes, wherein associations made in media are impressed upon viewers. Mobile sex-tech is technology used to enhance sexuality through information and connections with other people, such as through dating apps, among other activities (Gesselman et al., 2020). Sex-tech can be used for finding dating or sexual partners, and sending sexual images, videos, or message to others, known as sexting. In one study, an estimated 28.3% of individuals in Southeast Asia reported ever using sex-tech to find a sexual partner, and 60.1% had engaged in sexting, with approximately 45% having sent images and 28% having send videos (Gesselman et al., 2020). The anonymity of some of these platforms, as well as the independence with which they can be accessed by youth may lead to circumstances described by routine action theory, in which a lack of authority represents an opportunity for unhealthy action.

The rapid proliferation of internet and phone access also has vastly increased access to media that may contain biased framing of sexual violence, or sexually explicitly material (SEM), media demonstrating sexual acts, but not necessarily violent sexual acts (Owens et al., 2012; Peter & Valkenburg, 2016). An analysis of representation of sexual violence in German online news media found the perpetuation of rape myths and the portrayal of victims as weak and passive women (Schwark, 2017). In a study of English-language news articles from Pakistan, India, and the UK, it was found that gender-based violence messaging focused on rape, rather than sexual violence more broadly (Manzoor et al., 2023). Both studies demonstrate how prevailing ideologies normalizing sexual violence and minimizing different types of sexual violence may be perpetuated. While estimates of adolescent exposure to SEM vary globally (Peter & Valkenburg, 2016), studies in urban Vietnam confirm that large majorities of adolescents and emerging adults access SEM. A recent national study found that 84% of adolescents 15–18 years had ever been exposed to SEM (Nguyen et al., 2021), and a study of first-year male University students in Hanoi found that 41% had been exposed to media-based violent SEM in the prior 6 months (Bergenfeld et al., 2022a). Direct viewing of this content may provoke mimicry of violence by viewers, in alignment with sexual script theory. Notably, exposure to SEM may occur through passive intake or through active seeking of explicit materials. A 2011 analysis demonstrated that 83% of the top 20 Nielsen-rated adolescent television shows contained SEM (Neilsen Company, 2011), while a more recent content analysis of popular Western television shows watched by teens and young adults reached similar conclusions about the common nature of sexual violence and sexual abuse (Kinsler et al., 2019). Other avenues for passive exposure to SEM include video games, music, music videos, and films (Peter & Valkenburg, 2016). In studies of pornography, which is inherently explicit and often actively sought out, 88% of videos included physical aggression or violence towards women (Bridges et al., 2010; Foubert et al., 2011). These represent additional opportunities for priming of the association of sex with violence, with cultivation theory suggesting that repeated use increases the likelihood of adopting the beliefs presented in media.

Some of these paths of exposure to media on sexual violence also are tools for sexually violent behavior. Initially consensual sexting may quickly transition to unwanted exposure to sexual images or videos if one party does not seek consent. Informational and social media forums to share comments can become a vehicle for written sexual harassment of an individual. Images or videos shared consensually with a partner can be harnessed for blackmail or nonconsensual sharing via media. These direct, non-contact forms of sexually violent behavior are known as Technology Facilitated Sexual Violence (TFSV). They may include the distribution of explicit photographs and videos without consent, the sharing of unsolicited explicit content, and the use of online platforms for sexual harassment (Powell & Henry, 2019). In some western countries, lifetime TFSV exposure may be as high as 17% (Patel & Roesch, 2022), with higher prevalence among youth (Gámez-Guadix et al., 2015; Powell & Henry, 2019). Moreover, social media provides opportunities to facilitate in-person contact and non-contact sexual violence. Conversations on social media, both anonymous and not, may become an opportunity to convince someone to have an in-person meeting, either consensually or through manipulation of power dynamics; one party may have the intent of perpetrating sexual violence, physically, through nonconsensual documentation of sexual behavior, or through other means.

### Harms of Media/Technology on Violent Attitudes and Behavior

A growing body of evidence supports the harmful outcomes related to sexual material in media/technology, including the perpetuation of inequitable gender roles, rape mythology, negative self-worth, and sexually violent behavior. Several harmful outcomes also are associated with TFSV victimization, including technology use related to sex generally, such as sexting. In a Hong Kong-based study, individuals who took part in sexting had higher levels of body surveillance and shame than those who did not (Liong & Cheng, 2019); in some settings, youth may frame TFSV in dating relationships, such as demands to engage in sexting, as requests for “proof of love” (Fernet et al., 2023). Sexting itself has been associated with risky sexual behavior, substance use, depression (Gesselman et al., 2020) and self-harm among adolescents (Wachs et al., 2021).

In Western countries, exposure to SEM and especially violent SEM has been associated with more accepting attitudes about sexual violence and with sexually violent behavior (Rodenhizer & Edwards, 2019). In the global West and Asia, such exposure is associated with permissive sexual attitudes and gender-stereotypical sexual beliefs among adolescents (Peter & Valkenburg, 2016). Across studies, male study participants’ attitudes and behaviors regarding sexual and domestic violence were more strongly affected by exposure than females’ (Rodenhizer & Edwards, 2019). A recent meta-analysis found that greater overall media consumption was associated with higher rape myth acceptance (Hedrick, 2021), with general pornography, violent pornography, and sports media accounting for most of this association. In a recent longitudinal study in Vietnam, a dose–response relationship was observed between the frequency of exposure to violent SEM with non-contact and contact sexually violent (Bergenfeld et al., 2022a).

### Benefits of Media/Technology for Violence Prevention

Conversely, access and exposure to information on safe sexual behavior and the prevention of sexual violence can lead to positive outcomes among adolescents. Qualitative studies of women in the USA, Canada, and India have suggested that women who access non-violent sexual content associated this exposure with positive sexual exploration and development of sexual identity, opportunities for sex-positive education and exploration of readiness for sex, improved sexual connectedness in relationships, normalization of sexual desires, and improved acceptance of their bodies and sexualities (Arrington-Sanders et al., 2015; Attwood et al., 2018; Chowkhani, 2016; McKeown et al., 2018). Two qualitative studies conducted in urban Thailand and Vietnam found that adolescent girls use social media to develop their sexuality, express desires, and exercise sexual agency in settings where female expressions of sexuality are restricted and access to accurate sexual information is limited (Boonmongkon et al., 2013; Fongkaew & Fongkaew, 2016; Ngo et al., 2008). In a Hong Kong-based study, individuals who had participated in sexting had more comfort with nudity (Fernet et al., 2023), and sexting generally may contribute to increased emotional connection and satisfaction in relationships as well as freedom of sexual expression (Gesselman et al., 2020).

Social media also may provide a space for people to share sexual experiences and to seek support. In Hong Kong, “confessional” social media pages have enabled users to ask questions, seek advice around sex from peers, and receive peer support (Yeo & Chu, 2017). Social support, in turn, may indirectly reduce the risk of sexual violence victimization (Ybarra et al., 2015). In recent years, social media has been used strategically to disclose experiences of sexual violence and to provide social support to survivors (Alaggia & Wang, 2020), though some studies find that survivors of sexual violence do not reap the same benefits of public sharing of experiences as much as individuals who experienced less stigmatized trauma, such as a natural disaster (Delker et al., 2020). Also, the survivors of sexual violence may interpret and identify their experiences differently based on prevalence of sexual violence cases in the media (Newins et al., 2021).

Various forms of media have served as a tool to disseminate widely accurate and relevant information about sex and sexual violence to teen audiences (Todaro et al., 2018). Young people have cited increased comfort accessing information about sex online compared to other mediums (Lim et al., 2014), and reduced embarrassment for adolescents who are uncomfortable discussing sex with their parents (Lou et al., 2012). Qualitative research in Vietnam has found that parents would like accessible information about sex on the internet (Do et al., 2017). Numerous technology-based interventions relating to sexual violence exist, however, only a portion include content relating to violence prevention, rather than identification or survivor support, and those that do are largely focused on North America (Huang et al., 2022). In one systematic review of mobile sex-tech, which included 15 articles of technology-related interventions with sexual violence information, only 27% contained content on sexual violence prevention, and none contained information on the impacts of sexual violence (Huang et al., 2016). Nevertheless, two systematic reviews of tech-based interventions for intimate partner violence (IPV) found that IPV prevention in combination with access to telehealth services showed promise to reduce the risk of violence victimization (Anderson et al., 2021; El Morr & Layal, 2020), while a third did not identify any significant effects (Linde et al., 2020). Notably, the reviewed studies focused on interventions with women as potential victims, rather than men as potential perpetrators (Huang et al., 2022). While careful development is essential to prevent unintended consequences, such as increasing adherence to rape myths (Nicolla & Lazard, 2023), online programming in the U.S. and Vietnam have been successful in decreasing sexually violent behavior among university men (Yount et al., 2023b; Salazar et al., 2014; Yount et al., 2020). In a randomized controlled trial of an “edutainment” program to reduce sexual violence, program participants had increased knowledge of the illegality of sexual violence and increased victim empathy (Yount et al., 2022), through which Vietnamese men had lower odds of past-year sexually violence behavior after program participation (Yount et al., 2023b; Yount et al., 2020). This may demonstrate a pathway to reduced sexually violent behavior through the effective use of media with young people.

The aim of this paper is to elucidate the state of media usage relating to sexual violence among Vietnamese youth, according to educational and programmatic partners across Vietnam, using the research question “What is the perceived influence of social media on sexual violence among youth according to educators in Vietnam?” Further, we seek to describe the implications of media usage relating to sex and sexual violence, both positive and negative, and identify lessons and pathways to improve sexual health and sexual violence programming for Vietnamese youth. While literature is available on media, sex, and sexual violence globally and broadly in Southeast Asia, little of this research focuses on Vietnam specifically, and little from the perspective and framing of implementation of sexual violence prevention. This information may allow for effective, targeted programming and/or engagement in media to reduce sexual violence and promote gender equitable attitudes among youth.

Vietnam, located in Southeast Asia, is home to 96 million people, and 13.70% of the population are aged 15 to 24 years (General Statistical Office [GSO] of Vietnam, 2020). Fifty-four recognized ethnic groups are represented within the population of Vietnam, with 85% of individuals self-identifying as Kinh (Hiwasaki & Minh, 2022). Currently classified as a lower middle-income country, Vietnam’s population typically still resides in rural areas (70%) (World Bank, 2022); however, Vietnam has seen steady economic growth and diversification over the past 20 years, alongside declines in poverty (Do et al., 2021; Nguyen et al., 2020). Literacy is almost universal, and the gender gap in number of years of schooling is relatively narrow (United Nations Development Program [UNDP], 2022) though women less often participate in the workforce than men (69% v. 79%), and working women earn substantially less than their male counterparts (United Nations Development Program [UNDP]), 2022).

Nearly all individuals in urban and rural settings have access to electricity, and most (70%) of the Vietnamese population has access to the internet (Mobile Marketing Association, 2019). In 2019, there were 141 phone subscriptions per 100 inhabitants, and nearly all internet users are estimated to own a smartphone, such that over 80% of the population over age 15 is connected to online content (Mobile Marketing Association, 2019). Approximately 68% of the rural population own a smartphone, and as of 2019, was connected to the internet an average of 3 hours per day; of which 40% was spent on messaging apps in communication with others (Mobile Marketing Association, 2019).

As of 2019, 90% of young adults (18–29 years old) in Vietnam were using smartphones (Silver et al., 2019), while 72 million people (accounting for 73.7% of the population) use social media in Vietnam. Between January 2020 and January 2021 alone, the number of Vietnam’s social media users increased approximately by 10.8% (2021). Approximately 65% of Vietnamese youth (aged 16–30, *N* = 1200) use the internet daily; a study of social media use among Vietnamese youth engage with social media for an average of 4.3 hours a day and primarily use it to talk with friends and receive updated news (Doan et al., 2022). Commonly used applications include Facebook, YouTube, and Zalo, with interactivity on YouTube and Zalo being highest with news and entertainment accounts (Doan et al., 2022; Hanns Seidel Foundation, 2021).

Social media in Vietnam is currently regulated under the Law on Cyber Security (National Assembly of Vietnam, 2018), and the Decision 874/QD-BTTTT (2021) on the Code of Conduct on social media, to protect “the national security,” relating to “moral values, culture, and traditions of Vietnamese people” (Ministry of Information & Communications, 2021; My, 2022) While no specific definition of these terms is provided, Decree 15/2020/ND-CP outlines financial punishment of 10 million VND to 20 million VND ($500—$1,000) for violation of regulations on use of social media, including “promoting bad customs, superstition, lustful materials which are not suitable for the nation’s fine customs and traditions.” In sum, access to smartphones, online media, and networking apps are widespread in Vietnam, particularly among youth, and laws regulating access to online content are nascent.

## Method

### Participants

Recruitment strategies are described elsewhere (Yount et al., 2023a). In brief, a multi-pronged approach was used to identify key informants from universities, high schools, and civil society organizations (CSOs) who conduct programs related to sexual and reproductive health and rights. Once initial potential participants were contacted, the research team employed snowball sampling to diversify the participant pool, with consideration to institutional setting (university, high school, CSO), region of Vietnam in which the institution was located (North, Central, South), and gender.

Participants were invited via email to complete interviews until a total sample of 45 was achieved, with an even distribution of 15 participants each from university, high school, and CSO settings. Of 45 interviewed participants, 32 were invited to participate in focus groups, based on their knowledge of sexual violence programming. A total of 22 individuals agreed to participate in focus group discussions, resulting in four focus groups: one with high school teachers (*n* = 7), two with university lecturers (*n* = 6 and *n* = 3), and one with key informants from CSOs (*n* = 6).

### Measures and Procedure

We conducted a qualitative study of key informants from high schools, universities, and civil society organizations (CSOs) across all regions of Vietnam, which included in-depth interviews and focus group discussions, a mixed-methods approach which is useful for research that has multiple objectives (Hennink et al., 2020). Overarching findings from the parent study are presented elsewhere (Yount et al., 2023a). This analysis focuses on narrative segments related to the media, which was identified as a highly salient theme worthy of a separate, in-depth analysis.

In the parent qualitative study, the binational research team developed three guides for data collection. A semi-structured key informant interview guide contained open-ended questions about sexual violence among youth populations; gender and sexual norms among youth populations; causes, effects, and strategies to prevent sexual violence among youth; and factors influencing sexual violence prevention programming. Interviews were chosen to reduce social desirability bias, as recommended when discussing sensitive topics (Hennink et al., 2020). Two guides were developed for use in focus group discussions. Focus groups were selected for this objective as they allow for the identification of a range of perspectives and facilitate the justification of ideas (Hennink et al., 2020). First, a viewing guide elicited opinions and responses to the web-based sexual violence prevention program, GlobalConsent (Yount et al., 2022). The guide asked participants to rate on a five-point scale the feasibility and acceptability of program elements, and to elaborate upon their reasoning for the rating. The focus group facilitator collected the completed guides and used them in combination with a focus group guide to prompt discussion during the focus groups. The focus group guide aligned with the domains of the Consolidated Framework for Implementation Research (CFIR) (Damschroder et al., 2009). Questions were open-ended and assessed facilitators and barriers to sexual violence prevention programming, with prompts drawn from feedback in the viewing guide. This method was used to ascertain consensus, when possible. Of note, none of the data collection guides contained direct questions about social media, internet use, or technology-based sexual violence information dissemination other than reference to GlobalConsent. When participants raised these topics, facilitators asked follow-up questions to elucidate their opinions and experiences. All data collection forms have been previously published and are publicly available (Yount et al., 2023a).

All data were collected using audio calls through the Zoom videoconferencing system. Two research staff members trained in qualitative research methods from the Center for Creative Initiatives in Health and Population completed data collection. At the beginning of each interview or focus group, the staff members explained the purpose of the study and reminded participants that they would not be asking for any private or potentially sensitive personal information. All interviews and focus groups were recorded using the Zoom videoconference platform. Interviews lasted 45–90 min, and focus groups lasted 120–150 min. Upon completion of data collection, interview participants were compensated with $20 USD. Focus group participants were compensated $30 USD for viewing GlobalConsent and completing the viewing guide materials, and $20 USD for participating in the focus group discussion.

### Data Analysis

Data collectors saved digital recordings on a password-protected cloud-based research drive, and a professional transcription service transcribed all recordings verbatim. The research team verified random sections of the Vietnamese transcriptions against the original recordings. Verified Vietnamese transcripts then were translated into English, and a research team member proficient in the Vietnamese and English languages and cultures checked random segments of the English transcripts against the Vietnamese transcripts for accuracy of the translation and its meaning as intended in the original Vietnamese. Audio recordings were destroyed following quality checks of the written transcripts and translations.

Study team members analyzed the English transcripts using deductive and inductive techniques to identify initial themes and media-related sub-themes. Two doctoral-level study team members developed a codebook based on the interview guides and CFIR domains, inclusive of definitions, and revised it iteratively following repeated readings by other study team members. Team-based coding was used to code each transcript in MaxQDA.

All content related to media originally was coded using a single broad inductive code in the first round of coding. For this analysis, all coded segments related to media then were extracted and saved in a single document. Two graduate-level trained researchers independently reviewed this document to identify inductive sub-themes applicable to the data. The researchers then met to discuss the inductive sub-themes, reconcile discrepancies through inter-coder agreement, and to review the need for further theme identification. This latter step ultimately was not taken due to the high level of concurrence between the initially identified inductive subthemes. Inter-coder reliability was not calculated to avoid implied objectivity or undue precision (O’Connor & Joffe, 2020).

Finally, the coders created a salience matrix to visualize the presence or absence of each theme within each transcript, in order to qualitatively and quantitatively represent the salience of each them across all transcripts. Representative quotes for each sub-theme were identified to contextualize the findings with sector and gender of interview participants, and sector only for focus group participants, as statements were not individually identifiable in the focus groups. Participant characteristics are reported in Table 1.

**Table 1:** Interview and focus group participants, Vietnam

	High school teachers (*N* = 15)	University lecturers (*N* = 15)	Community service organization members (*N* = 15)	Total (*N* = 45)
*Interview participants*				
Sex				
Female	11 (73.3%)	11 (73.3%)	13 (86.7%)	35 (77.8%)
Male	4 (26.7%)	4 (26.7%)	2 (13.3%)	10 (22.2%)
Region				
North	9 (60.0%)	5 (33.3%)	10 (66.7%)	24 (53.3%)
South	2 (13.3%)	8 (53.3%)	3 (20.0%)	13 (28.9%)
Central	4 (26.7%)	2 (13.3%)	2 (13.3%)	8 (17.8%)

	High school teachers (*N* = 6)	University lecturers (*N* = 9)	Community service organization members (*N* = 7)	Total (*N* = 22)
Focus group participants				
Sex				
Female	5 (83.3%)	8 (88.9%)	4 (57.1%)	17 (77.3%)
Male	1 (16.7%)	1 (11.1%)	3 (42.9%)	5 (22.7%)
Region				
North	4 (66.7%)	5 (55.6%)	3 (42.9%)	12 (54.5%)
South	2 (33.3%)	3 (33.3%)	1 (14.3%)	6 (27.3%)
Central	0 (0.0%)	1 (11.1%)	3 (42.9%)	4 (18.2%)

Reproduced from Yount et al. (2023a), with permission from the authors

## Results

### Accessibility and Quality of Information about Sex and Sexual Relationships

Many community partners, including more than half of participants employed as university lecturers or CSO affiliates, noted that media is highly accessible to young people in Vietnam, particularly through the internet, such that information about sex and sexual relationships can be easily found. Further, according to many participants, students seek out information independently, rather than rely upon schools to provide information. According to one participant, “Young people now have access to information easily, so they can learn things themselves. When we …distribute free condoms to advocate for safe sex, it comes to my surprise that students actually know about condoms” (University Lecturer, Woman). Another participant clarified that easy access to information about sex was generally a benefit of media access, specifically for young women, “*…* nowadays, I think with the age of technology development, it would be easy for [young people] to look for information to help protect themselves [from unwanted pregnancy]” (University Lecturer, Woman).

A few participants described this high general access to information about sex as empowering for young people in Vietnam. One participant stated, for example, that “When they [the youth] need information, they are very proactive and find it very quickly themselves” (University Lecturer, Woman). However, several participants, and particularly participants affiliated with CSOs, expressed concern that the quality of available information on sex and sexual relationships is variable, and some “unregulated” available content may be inaccurate and may lack the comprehensiveness that adolescents need. One participant shared, “In recent years, access to the internet makes it easy for people to retrieve a lot of information, including unregulated ones, especially for adolescents and youth. People are not fully informed” (CSO Affiliate, Woman). Elaborating on this potential for youth to gain incomplete knowledge through internet sources, another participant noted, “…I can see that the young generation has learnt on their own via the internet … even though the young have somewhat an understanding about safer sex, their source of information is not adequate and they still lack orientation” (University Lecturer, Woman). This lack of orientation was seen as an important barrier to the practice of safe and consensual sex.

A few participants expressed additional concern about the volume and appropriateness of information available online, given the lack of formal sex/sexuality education, and given the willingness of students to actively see information themselves: “…sometimes the information is too much for them. …there really isn't anyone to teach them the skills to say no. So really they mostly depend on their instinct or information that they find out on their own rather than having orientation” (University Lecturer, Woman). When the information availabe online is “too much,” according to some participants, students may experience potentially harmful outcomes. Participants discussed how the abundance of ostensibly tempting, unregulated, and confusing information could overwhelm youth seeking information, with one participant explaining, “[online] we also receive many opinions, different kinds of feedback, even some pages, organizations, and activists …. I think it [creates] a rather chaotic environment …[Youth] can feel confused since they don't know which side to take, they are not sure which side is the right one” (CSO Affiliate, Woman).

About one third of participants from high school and university settings attributed this confusion to the lack of up-to-date “official” information, with one lecturer noting, “they have too much information and couldn’t find any official information,” calling the available official sources of information “extremely limited and old school” (University Lecturer, Woman). Many paricipants believed such official information was vital to provide young people with accurate information about sex. A CSO-affiliated participant shared,

Now it's easy, everyone has a smartphone. Google does not charge, so just google it. ... However, [youth] are not sure which document is official and standard. What they need the most is the most up-to-date materials with information and knowledge constantly updated. The source of information must be official and in accordance with the standards. (CSO Affiliate, Woman*)*

Despite substantial agreement across participants that youth needed more access to “official” information about sex, the meaning of “official” in the context of information on sex in the media varied. One participant distinguished information from medical sources and that from the government and schools, with the latter sources being indicated as preferrable, while medical sources, such as hospitals, may contribute to the potentially overwhelming, “unregulated” information online: “There are also many video clips or guideline on other social networks… Some hospitals also post these contents, both foreign and Vietnamese. [It’s] available, but maybe there’re too many, with no sources from the Government or schools.” (CSO Affiliate, Woman)

Other participants echoed the idea of “official” sources originating with the government, with one stating, “…online, I have not seen much. Besides, I think there is no official training program [on this topic] by the Ministry of Education yet” (CSO Affiliate, Woman). The absence of an official training program complicated the role of schools and universities, who were noted as potentially playing a significant role in the dissemination of information, but only once it exists: “And when the information is official, it will be shared by the school, the university, or the student community. It is also considered an official channel” (CSO Affiliate, Woman). Only one participant discussed non-governmental organizations as a source for “official” information: “If we talk about official sources of information, it is definitely the type of absolutely official sources like UNWOMEN or …multinational organizations working on gender. They provide much more credible information” (CSO Affiliate, Woman).

### Media Depictions of Sexual Activity and Normalization of Sexual Violence

Some participants noted the lack of official information regarding sex and sexual relationships as intersecting meaningfully with narrow, sensational depictions of sexual violence and the normalization of sexual coercion, non-consent, and sugar-daddy relationships in the media. These depictions were described as occurring in the news media, television, and in online media content, including but not limited to pornography. One participant shared that the news media was not adept at conveying the complex nuances of sexual consent, arguing that this source of information was “unable to distinguish at a more delicate level, that things may start out as consensual, but could become non-consensual later” (University Lecturer, Woman). Only extreme cases of sexual violence were cited as worth covering by news media, masking other forms of sexual violence. As a focus-group discussion participant explained, “[the news media] only care about the major events … if there is a case where the students …make a girl to drink and it leads to a rape, and that spreads among students… [the media] will immediately jump in to investigate and interview” (University Lecturer). Further, according to another participant, the news media’s presentation of information on cases of sexual violence often ignore the voices and experiences of women and experts in the field.

Some participants also described the silencing of women’s experiences relating to sex and sexual violence as common in the movies. Some participants described movies from Southeast Asia as reinforcing rigid gender stereotypes, including women’s passivity and submission to masculine coercion, as well as the normalization of non-consent. A CSO affiliate shared, “…for example, in Chinese and Korean dramas, we may see scenes where the female character doesn't like it, but the male keeps kissing, and then, in the end, those two have a sweet night. I feel such things are injected into girls’ heads” (CSO Affiliate, Woman). One high-school teacher corroborated the above depiction of sex in Southeast Asian movies in this way:

…in Korean/Chinese movies or love novels that the young usually read and watch, the main characters typically force their partner into having unwanted sex or physical contacts, such as hugs or kisses. Consequently, the readers form a notion that it is okay for the male to force such activities, and the female does like it. (High School Teacher, Woman)

Reinforcing the normalization of sexual violence and non-consent in mainstream movies available to Vietnamese youth, some participants, particularly university lecturers, discussed how pornography contains harmful depictions of sexual activity that they believe drives a desire among men to imitate, including violent behaviors or behaviors that would make female partners uncomfortable. As demonstrated by the experiences of one university lecturer, exposure to pornographic material was expansive, and occurred from a young age:

…men from old to young have seen this type of [sexually explicit] film and are influenced by the erotic and sadistic elements in the film. These factors often stimulate the curiosity of men and make them want to try more. Young people are especially curious about this issue. I once did a project to provide computers for elementary schools …after 6 months of operation, when I accessed the search history, there were many pornographic websites in it. The 5th graders at that school watched sex movies. Even though they did not type the right words, they still watched and even discussed the contents of the movies. (University Lecturer, Man)

Some participants believed that youth generally were not aware that violent actions portrayed in such movies would be considered sexual violence, but rather normal sexual encounters that should be used as a guide. One university lecturer stated, “…young people think that if they do the same thing [as pornography], they will be professional, and this mindset causes young people to commit acts of sexual violence that they themselves do not know,” (University Lecturer, Woman). Several participants emphasized that the risk of imitating sexually explicit material, and at times violent sexually explicit material, was heightened because official information about healthy sexual relationships was lacking and so could not counter adolescents’ interpretations of SEM as normal forms of sexual interaction.

Concurrently, some participants, and particularly high school teachers, perceived a rise in portrayals of transactional sex in online media that capitalize on gender-stereotypical roles and economic power imbalances, particularly manifesting in “sugar-daddy” relationships among young people. One CSO affiliate shared, “… the most current trend [on dating apps] is sugar-daddy and sugar-mommy, which is why people nowadays joke that formerly, you could only purchase a single ticket for one-night stands, but now you can purchase a multiple-round ticket.” (CSO Affiliate, Woman). According to one high school teacher’s experience, sugar-daddy and sugar-mommy relationships were normalized in online media: “Last year, for example, there were so many cases of sugar babies—sugar daddies. They even made short clips to gain views and likes and created videos to post on YouTube to make money.” (High School Teacher, Woman). Another participant from a CSO contrasted her own personal experiences with those of youth only a few years younger: “My age is pretty close to them [but] when I was their age, those types of [transactional] relationships weren’t that common around me and not on the media either. Now it’s much more popular in the media…. These things are normalized because it’s common among young people” (CSO Affiliate, Woman).

### Mixed Consequences and Opportunities from Young People’s Engagement with Media

To many focus group and interview participants, including many of high school teachers and university lecturers, increased access to and interaction with media, the lack of reliable information on sexual relationships and sexual violence, and media portrayals and normalization of non-consensual and violent relationships led to increased risk of sexual violence victimization among youth. The accessibility of communication with unknown persons provided by internet access was viewed as facilitating these cases of violence by providing opportunities for youth to be taken advantage of. A CSO affiliate spoke to this point, saying, “They [youth] often use communication apps to date or *have sex* (slang). Even those children of 15, 16 age… showed their bodies online as requested by older men” (CSO Affiliate, Man).

Several participants elaborated that youth they were familiar with also were at risk of non-consensual recording and distribution of content. Over one-third of high school teachers discussed these risks, and a participant who worked with a youth-centered CSO detailed them, saying, “…some people lure students into the toilet to secretly film them. … [some are] paying students to go to their living space to perform sex, then record clips…. On paid sex viewing websites, many videos with private sex scenes are posted to get money" (CSO Affiliate, Man). This non-consensual sharing of explicit sexual material was not limited to circumstances of non-consensual recording but could also originate in the consensual sharing of videos or images among youth, or between youth and adults. This content was described as potential material for blackmail and manipulation: “Four cases that I handled last year had to do with online erotic messaging via Zalo or text messages: boys and girls, they exchanged erotic images, but the boy saved those images and used them to blackmail the girl." (University Lecturer, Man) According to some participants, they didn’t believe youth understood the risks of content sharing, even to a single person: “The images uploaded on the Internet can be viewed by millions of people. People do not think of it as a danger to themselves. There are cases where private pictures/videos are spread out right in the school. Students did not think that it could be able to be exposed.” (CSO Affiliate, Man).

Conversely, some participants acknowledged benefits to increased access and exposure to media. The internet provides forums where youth who had experienced sexual violence could connect with other survivors, fostering social support: “Nowadays, the young can share their story with some groups or forums on the Internet. …though talking about their situations on the Internet didn’t get them the professional support they needed, they did receive a certain level of empathy and emotional support” (CSO Affiliate, Woman). Another participant noted that the internet provided a layer of privacy that could not be achieved through in-person contact, easing feelings of embarrassment or discomfort in seeking support: “I think they would talk to their friends… [but] talking [in person] could be embarrassing, and they would be afraid that someone might overhear them, so I think they would send messages over Zalo, Facebook.” (University Lecturer, Woman)

Furthermore, social forums on the internet were seen by some participants as providing space for young people to ask questions, to be exposed to different opinions, and to gather a variety of perspectives to inform themselves better: “In this era, information is very accessible. However, what really helps is a space where they feel safe to share their views on this topic. And after sharing, they can also listen to other people's opinions to conclude what is right, what is reasonable, what is not reasonable, and what is needed to be changed?” (University Lecturer, Woman).

### Media as a Tool for Sexual Education and Sexual Violence Prevention

Despite participants’ perceptions of the mixed outcomes related to high accessibility of heterogeneous information about sex and sexual relationships and the high use by young people of media for this information, many participants noted that media was a powerful and important tool for disseminating information about sex and sexual violence and for engaging youth. One high school teacher shared, “…with the advance of technology in today's society, we can create websites, Facebook pages, or TikTok channels…to share information about sexual violence and ways to prevent it. I think this is a way for the young to access reliable sources of information more easily” (High School Teacher, Woman). *How* information is presented in media also was discussed, with some participants highlighting that media can be a tailored medium of information to youth. The flexibility of media as a medium, and particularly online media was noted as a major facilitator or tailoring information. An CSO-based participant shared: “We can run several media projects that propagate sex education content such as talk shows, minigames, etc.… There are certain levels of flexibility for communicating with students of this age, especially in a time when sex education is still something we are aiming at" (CSO Affiliate, Woman). Finally, some participants, particularly those affiliated with CSOs, noted a key feature of media as a potentially effective tool was the ability to reach large populations:

The biggest advantage of youth groups like us, or young NGOs working in the social field, we know about social media, and we use it quite proficiently… Hence, I feel that is our huge advantage, especially if we want to spread the knowledge about sexual violence on the media and social networks. That's quite true because, among our 150,000 followers, 78 - 80% of them are young people aged from 18-20 years old. (CSO Affiliate, Woman)

Other participants echoed these sentiments, with one university lecturer suggesting that existing institutional resources could be leveraged, saying, “I think the best method would be using websites, the ones that are familiar to students, or pages of the student community " (University Lecturer, Woman). This strategy was cited as providing the sense of an “official” channel of information, and more clearly delineating between “official” and “unofficial.” Other partnerships also were discussed, including working with media channels: “I think we can also use the help of media news channels and authorities to spread more awareness and attract attention to the topic of sexual violence.… By doing this, the young do not have to actively learn about it … their mindset about sexual violence can be formed unconsciously" (High School Teacher, Woman). By these means, participants recognized media as potentially facilitating norms change through the efforts of activists and educators, underscoring the perceived power of media as a tool, “…not just to inform people, but also advocate for public opinions.” (University Lecturer).

Despite the numerous positive features of media as an educational tool, a few participants noted limitations. One participant shared, “I can't force them to visit only this page, go to that page, or tell them that they can only read materials related to learning and mustn’t watch movies. That is very difficult" (High School Teacher, Woman). Another participant pointed out that, despite the accessibility of information on the internet, neither access to nor use of the internet to seek out information were universal, potentially leaving vulnerable populations out of media-based programming:

Not everyone can have access to social media, or to the internet. Not everyone has time…. For me, the people who don't … have the conditions to do it are the people we need to approach most. Because those people are people who don't have much access to the mass media, to both information and knowledge sources on gender and sexual violence. (CSO Affiliate, Woman)

Even with these limitations, several of participants acknowledged that media already has shifted norms in Vietnam. One participant said of the youth with whom they work, “With the influence of social media such as YouTube, Tiktok, or Facebook, young people view sexual intercourse at this age as something normal. They think it is no longer a shame as in older times; they even openly share about it instead of keeping it a secret as before" (CSO Affiliate, Woman). Another key informant shared the impact of media on independent decision-making among youth, stating that media has given youth freedom through access to information. Finally, one participant commented on the increasing globalization allowed by media, and the profound impacts of exposure to different ideas:

I believe that the power of media and other means of connecting people, such as social networks, is quite large… There is also the openness of social media, where Vietnamese adolescents are more exposed to Western culture through concepts like freedom of expression and self-expression. Furthermore, girls are no longer constrained by the old concept of virginity. Boys, on the other hand, have more opportunities to study abroad and interact with people from different cultures, resulting in more cultural exchange. There are also an increasing number of reality shows on television about love and romance. As a result, they have a wealth of resources at their disposal to learn more about sex. (CSO Affiliate, Woman)

Leveraging of these resources, participants agreed, could be used to promote sexual health and decrease sexual violence among Vietnamese youth, empowering adolescents to shift social norms and promote increased gender equity.

## Discussion

### Summary and Interpretation of Findings

In interviews and focus groups regarding sexual violence prevention among adolescents in Vietnam, high school teachers, university lectures, and affiliates of youth-focused CSOs expansively discussed the role of media and technology in the context of sexual violence and sexual education for Vietnamese youth. Primarily, interview and focus group participants expounded upon the high availability of access to media through technology, and particularly media relating to sex. However, the information presented in this media varies widely, according to participants, with some media that is incorrect or inappropriate for youth, and few available sources of information that were classified as “official.” Respondents shared that the available media—including news media, informational websites, social networking platforms, video streaming platforms, and mobile applications—depict sexual activity that is coercive or violent, normalize transactional sex, reinforce normative beliefs about inequitable gender roles, and prompt mimicry of sexual violence.

Given the high prevalence of youth information-seeking about sex through media (Buhi et al., 2009; Santor et al., 2007), including in Southeast Asia (Gesselman et al., 2020; Ngo et al., 2008; Nguyen, 2007) and Vietnam, specifically, the availability of accurate and appropriate information on sex and sexual violence is imperative to the education of youth globally. In line with behavioral health theories that integrate mimicry and observational or social learning, such as sexual script theory (Wiederman, 2015) and social cognitive/social learning theory (Bandura, 2001; Bandura & Walters, 1977), participants stated that youth they were familiar with imitated the sexual situations and actions they viewed in media, including violent sexual acts without the consent of their partner. This echoes previous findings of the role of observational learning in the contexts of media, violence, and sexual activity, previously identified associations between exposure to SEM, sexual attitudes, and gender-stereotypical sexual beliefs in the global West and Asia (Gesselman et al., 2020; Peter & Valkenburg, 2016), and recent findings on a dose–response relationship between SEM and sexually violent behavior among young men in Vietnam (Bergenfeld et al., 2022a). Participants stated that non-consent was normalized in movies and television, passively reinforcing the violent and coercive sexual behaviors, while news media both sensationalized and neglected to portray the nuances of sexual violence and consent. According to cultivation theory, repeated exposure to portrayals and normalization of sexual violence may promote greater acceptance of and desensitization to violence (Morgan et al., 2014), which may provide insight into why some participants reported that youth did not know that nonconsensual sexual acts were in fact sexual violence.

Participants also discussed the implications of this increased access to media relating to sex, with both negative and positive outcomes delineated. Media was cited as increasing opportunities for sexual violence in two main ways. First, youth were put at risk through connecting with unknown people via messaging or networking sites, leading to vulnerable in-person meetings. Indeed, almost 30% of individuals in Southeast Asia are thought to have used sex-tech for finding sexual partners (Gesselman et al., 2020), indicating willingness to put oneself in situations that may be conducive to sexual violence. Second, youth were put at risk through the sharing of their own sexually explicit media with others, either privately or publicly. An estimated 60.1% of individuals in Southeast Asia report sending sexually explicit messages, including images and videos (Gesselman et al., 2020), which have the potential to then be shared beyond the original recipient without consent. Non-sexually explicit photos posted on social media were also described as a pathway for sexual harassment or TFSV.

By contrast, media also provided forums for learning, exploration, and social support, which participants noted is promising for sexual education and prevention of sexual violence. Social media and blogs were cited as potential sources of diverse opinions and experiences, corroborating previous findings of young women in Southeast Asia using the internet to explore and develop sexual identities and gain accurate information about sex (Boonmongkon et al., 2013; Fongkaew & Fongkaew, 2016; Ngo et al., 2008), and seek advice and social support about sex and sexual violence (Alaggia & Wang, 2020; Yeo & Chu, 2017). The significant portion of Vietnamese young adults on social media (Silver et al., 2019) also marks this as an ideal pathway for education and prevention of sexual violence, as identified by interview and focus group participants.

Interestingly, while participants recognized the diversity of the sources of information on the internet and the impossibility of "forcing" students to view "official” channels, they did not discuss the importance of media literacy education, which may help students to identify the useful/good vs. harmful information. It may be important to emphasize students as agents for change—once they are equipped with the knowledge and skills to analyze media sources with discernment and more accurate knowledge of sexual violence and consent. Media literacy education in high school and university contexts may offer a normatively acceptable pathway to increased critical analysis of sexual and sexually violent content in media, and may not face the same barriers that have been outlined to implementing sexual violence prevention education in Vietnam (Yount et al., 2023a). This strategy may complement ongoing violence prevention efforts, particularly in high schools given that average educational attainment is projected to exceed 12 years in Vietnam among children of school-entry age (United Nations Development Program (UNDP), 2022). Such programs that have been successfully implemented in the United States may be appropriate for adaptation to the context of Vietnam (Scull et al., 2018, 2022).

### Limitations and Strengths of Analysis

Some limitations and several strengths of this analysis are notable. First, qualitative research is not generalizable but generates salient themes that may be explored more systematically in future surveys involving representative samples of young people. Second, the data are perceptions of the behavior of young people from high-school teachers, university lecturers, and CSO affiliates and should not be interpreted as the actual behavior of young people. Relatedly, there is a possibility of polysemy in interpretation of media, wherein messaging may be interpreted semantically differently by different individuals (Ewoldsen et al., 2022), with implications for processing and actions taken following viewing. Educators may not only interpret media they see differently from their students, but students in one cultural group, class, or educational environment may view or interpret media differently from others, limiting the interpretations educators may be privy to. Only comprehensive inclusion of diverse educators—for the circumstances of this analysis—or youth can capture these multiple interpretations. While significant efforts were made to engage educators from across geographic regions and genders, these efforts may not be sufficient to capture all educator interpretations, let alone those of youth. Despite these caveats, the sample of participants is highly diverse, representing men and women living in urban and rural areas and key informants from diverse youth-serving institutions across all regions of Vietnam. Moreover, the participants in the study, because of their profession and high degree of interaction with young people, are important key informants to query, as knowledgeable adults from their own vantage point. Finally, the team used theory on media and violence to inform a nuanced interpretation of the data and its alignment with results from prior empirical research. The findings provide important insights about possible next steps to understand and to address young people’s use of the media and the diverse and sometimes countervailing ways in which it may help or harm young people’s encounters with sexual violence.

### Implications for Research and Sexual Violence Prevention Programming

The findings from this analysis are a strong call for more research among youth in Vietnam, especially surrounding the needs for comprehensive sex education and TFSV. The increased prevalence of TFSV also warrants including this outcome in measures of sexual violence, such as those used in population-based surveys. Thus, large-scale surveys among high-school studies and university studies to document in representative school-based samples the various ways in which media and violence intersect, and at what developmental ages, would provide critical groundwork for developing educational programming that meets the most salient needs at each developmental stage. Expanded research is needed on youth media literacy and the needs of educational systems to facilitate implementation of sexual violence programming (Yount et al., 2023a). Finally, given increasing access among youth globally to media-related technology and the internet, more work is needed to understand the implications of this access for sexual violence and sexual health, particularly among youth in low- and middle-income countries.

From the perspective of sexual health and sexual violence programming, the findings from this analysis are suggestive of some common and some age-specific needs of young people in Vietnam. First, there is a clear call for official, science-based curricula on sex and sexual violence that is developmentally tailored to high school and university students. A stronger need may exist for comprehensive sexuality education at the high-school level, including education on healthy relationships and media literacy. It may be beneficial to incorporate international standards, such as those suggested in the UNESCO Comprehensive Sexuality Education Implementation Toolkit (UNESCO, 2023), into newly developed curricula. Existing effective programs delivered through technology to university students (Yount et al., 2022) may be adaptable for the context of high school students, facilitating continuous sexual health and sexual violence education through high school and university. At the university level, there may be a more salient need for sexual violence prevention programming that educates adult students about the nature and scope of sexual violence, the importance of obtaining active consent for sex, the role of (media disseminated) gender norms in perpetuating myths about rape, masculine privilege, and ideas that are harmful to healthy sexual relationships (Bergenfeld et al., 2022b, 2022c). Notably, peers themselves are an important source of norms about sexual violence alongside media; as such, comprehensive sexual violence prevention programming must address both media- and peer-related risk factors for sexually violent behavior (Yount et al., 2022). Furthermore, there is a need for education at both the high-school and university levels about the safety of online dating and social networking so that students are better informed about the risks at the outset of their engagement with online social-network and dating aps. Finally, there is a need for comprehensive education about the types of sexually explicit material that may heighten risks of sexually violent behavior for both groups, given the high prevalence of exposure at a young age in Vietnam (Bergenfeld et al., 2022a).

### Conclusions

The rise in availability and exposure to media among youth globally and in Vietnam has raised new educational needs on sexual violence prevention and sexual health information. New and adapted curricula, with age-specific programmatic elements, may help to mediate the impacts of media on perpetration of violence.
